# Involvement of Pathogenesis-Related Proteins and Their Roles in Abiotic Stress Responses in Plants

**DOI:** 10.3390/biom15081103

**Published:** 2025-07-30

**Authors:** Yilin Zhu, Fei Gao

**Affiliations:** College of Life and Environmental Sciences, Minzu University of China, Beijing 100081, China; 22011910@muc.edu.cn

**Keywords:** pathogenesis-related protein, abiotic stress, chitinase, thaumatin, drought stress, low-temperature stress

## Abstract

Plant pathogenesis-related (PR) proteins are a large and diverse family of proteins with antimicrobial activity, often induced by pathogen attack. Traditionally, PR proteins were thought to mainly participate in plant defense mechanisms against biotic stress. However, in recent years, increasing evidence has shown that these proteins also play important roles in the response to abiotic stress in plants. In the present review, we provide a summary of the latest findings on PR proteins and focus on their response to various abiotic stresses, the mechanism by which PR proteins are activated by external and internal signals, and their biological functions in plant responses to abiotic stresses. In addition, the existing challenges and future applications are also summarized, aiming to provide a reference for further research on PR proteins in the context of plant physiology.

## 1. Introduction

It is imperative to enhance the resilience of crops to abiotic stresses to meet the escalating global demand for food. Abiotic stresses, including drought, high salinity, heat, low temperature, heavy metals, ultraviolet light, and waterlogging, are prevalent unfavorable conditions for crop cultivation. Crops frequently exhibit diminished yield and compromised product quality under abiotic stresses. It is evident that climate change, characterized by rising global temperatures, will lead to an increased frequency of extreme weather events, such as heat waves and droughts, across the globe. Despite the considerable efforts of scientists to engineer abiotic stress tolerance in economic plants, there remains a paucity of robust crop varieties that demonstrate substantial resistance to abiotic stresses. In recent years, the field of plant research has seen a surge of interest in the study of the roles of plant pathogenesis-related (PR) proteins in plant responses to abiotic stresses. These proteins were originally recognized to be induced by a variety of abiotic stresses. Moreover, transgenic plants overexpressing *PR* genes exhibit significant abiotic stress resistance. To enhance the comprehension of the biological functions of PR proteins in relation to abiotic stress tolerance in plants, a comprehensive review of the existing literature was conducted. This review outlines the classification of PR proteins and reported abiotic stressors inducing *PR* genes and proteins, summarizes the impact of changes in *PR* genes expression on plant abiotic stress tolerance and the possible regulatory mechanisms underlying the response of PR proteins to abiotic stress, and points out the existing challenges and future applications, with the aim to provide a reference for further research on PR proteins in the context of plant physiology.

## 2. Overview of PR Proteins

PR proteins refer to a broad category of proteins induced by various biotic or abiotic stresses in plants [1]. They were initially detected in tobacco leaves infected with tobacco mosaic virus (TMV) [2]. The accumulation of PR proteins in both infected and non-infected parts of plants has been shown to induce a hypersensitive reaction (HR), cause local cell death, and result in the development of systemic acquired resistance (SAR) in the plant, thereby limiting the spread of pathogens [3]. The molecular weights of PRs range from 6 kDa to 43 kDa, and these proteins are characterized by their resistance to heat and proteases, as well as their solubility at low pH (<3) [4]. Currently, PR proteins are classified into 17 distinct families (PR-1 to PR-17) based on their plant origin, serological properties, and amino acid sequence homology [1]. These families includes one PR-1 family with unknown biochemical properties, one β-1,3-glucanase family (PR-2), four chitinase families (PR-3, PR-4, PR-8, and PR-11), one protease inhibitor family (PR-6), and one specific peroxidase family (PR-9), as well as the thaumatin-like protein (PR-5) family and ribonuclease-like protein (PR-10) families. The subsequent introduction will provide a brief overview of the 17 PR families, accompanied by a summary of their structural characteristics and primary biological functions.

### 2.1. PR-1 Proteins

The first member of the PR-1 family was the earliest identified PR protein [2], and its presence was subsequently detected in monocotyledonous and dicotyledonous plants. PR-1 proteins possess molecular weights ranging from 14 to 16 kDa [5], and they are classified into two groups: acidic and basic. Acidic PR-1 proteins are secreted into the extracellular space, while basic PR-1 proteins are predominantly localized within the vacuole [6]. All PR-1 proteins contain a CAP (cysteine-rich secretory protein, antigen 5, pathogenesis-related 1) domain. This CAP domain is folded into a distinctive α-β-α sandwich structure, which generally consists of four α-helices and a four-stranded β-sheet, stabilized by conserved disulfide bonds. The structure of the CAP domain determines the function of PR-1 proteins in plants, thereby conferring them antifungal properties [7]. It has been demonstrated that PR-1 proteins exert their antiviral activity by impeding the binding of viral capsid proteins to plant receptor molecules. This inhibition occurs through a multifaceted mechanism that involves the attack on pathogens, the degradation of cell wall macromolecules, and the prevention of viral capsid protein binding to plant receptor molecules [8]. Beyond their role in biotic stress responses, PR-1 proteins demonstrate responsiveness to various abiotic stresses, including drought, elevated salt levels, low temperatures, high temperatures, and heavy metals [9,10,11]. Furthermore, PR-1 proteins have been identified as playing a pivotal role in a variety of metabolic pathways, including the MAPK signaling pathway and phytohormone signaling [12].

### 2.2. PR-2 Proteins

PR-2 proteins are members of the glycoside hydrolase superfamily, specifically the β-1,3-glucanase subfamily. PR-2 functions as a catalyst, facilitating the intramolecular cleavage of the 1,3-β-D-glucosidic bond, thereby generating single β-1,3-glucan units. The PR-2 proteins are characterized by the presence of a glycoside hydrolase domain and a secreted signal peptide (NTS). PR-2 level is induced by pathogen infection, plant hormones such as salicylic acid (SA) and abscisic acid (ABA) [13], as well as by low temperatures. β-1,3-glucan is an important component of fungal cell walls. PR-2 proteins directly hydrolyze β-1,3-glucan in fungal cell walls, causing hyphal deformities and lysis, and exhibit antifungal activity both in vitro and in vivo [14]. Moreover, PR-2 degrades the cell walls of pathogens to produce various types of oligosaccharides, which can promote the release of defense elicitors from plants and activate plant immunity [15]. Many pieces of evidence indicate that PR-2 proteins have been implicated in the metabolism of plant callus, the regulation of plant development, and the intercellular transport of hormones and RNA [16,17]. Other studies have shown that PR-2 proteins participate in plant response to various abiotic stresses including drought [18], low temperature [19], osmotic stress [20], salt, and heavy metal stresses [21].

### 2.3. PR-3, PR-4, PR-8, and PR-11 Proteins

The PR-3, PR-4, PR-8, and PR-11 families are all distinct classes of chitinases. The molecular weights of these proteins range from 25 to 36 kDa, with the majority falling within the glycoside hydrolase GH18 and GH19 families. Chitinases were classified into seven distinct classes based on structural characteristics, substrate specificity, catalytic mechanisms, and sensitivity to inhibitors [22]. Class I chitinases are basic proteins that are secreted to vesicles and plasmodesmata. They have a C-terminal propeptide that helps to target vacuoles, and N-terminal chitin-binding domains (CBDs) that are proline- and glycine-rich. Class II chitinases are acidic proteins that are secreted to the plasmodesmata and they lack a CBD domain. Class III chitinases are acidic proteins that are secreted into vesicles and apoplast, and these proteins contain two N-terminal LysM domains, which contribute to antifungal activity and lack a CBD domain [23,24]. Class IV chitinases are also acidic proteins that are secreted into the apoplast, and their N-terminus contains Hevein domains that can bind to chitin [25]. Class V chitinases are secreted into the apoplast and have a C-terminal extension and two CBD domains [26]. Class VI chitinases are acidic proteins secreted into vesicles and the apoplast. Class VII chitinases do not have any CBD [23,27]. The PR-3 family comprises the class I/II/IV/VI/VII chitinases, which are characterized by the presence of a common CBD domain and a helical structure with the catalytic domain. The PR-4 family and the PR-3 family, despite their classification as class I and II chitinases, exhibit significant structural divergences. PR-4 family proteins contain three intramolecular disulfide bonds and a conserved C-terminal structural domain, BARWIN, which is implicated in antifungal defense [28]. The PR-8 family is classified as class III chitinases, with acidic and basic isoforms. The PR-8 family exhibits both lysozyme activity and the potential for antibacterial activity. The PR-11 family, classified as class V chitinase, lacks the typical CBD domain [24]. In general, PR-3, PR-4, PR-8, and PR-11 proteins have been shown to inhibit fungal spore germination, combat diseases by degrading β-1,4-chitin chains in fungal cell walls and exoskeletons of insects [29,30,31], and exhibit antiviral activity [24,32]. Beyond their antifungal and antiviral functions, chitinases have also been implicated in plant responses to abiotic stresses such as drought [33], low temperatures [34], heavy metals [35], and high temperatures [36].

### 2.4. PR-5 Proteins

PR-5 proteins are thaumatin-like proteins (TLPs) [37], initially identified in tobacco leaves infected with TMV [38]. Several members have been reported to possess endo-β-1,3-glucanase activity and α-amylase inhibitory activity [39]. The PR-5 proteins are divided into two classes: one class (large TLPs) with a molecular weight of 21–26 kDa and with 16 conserved cysteine residues. The molecular weight of the other group (small TLPs) has been determined to be between 15 and 18 kDa, and small TLPs contain 10 cysteine residues [40]. The core conserved domain of PR-5 proteins is the thaumatin fold, a conserved domain that consists of two antiparallel β-sheets arranged in a “β-sandwich” conformation. This structure typically encompasses approximately ten β-strands interconnected by α-helices and loop regions [41]. PR-5 proteins are involved in various physiological processes, including plant development, secondary wall synthesis [42], and developmental regulation and ripening of fruits [43]. Furthermore, PR-5 proteins are involved in auxin signaling pathways [44]. PR-5 proteins possess antimicrobial enzyme activity [45] and are also regulated by stress factors such as high salinity [46] and viral or bacterial infections [47].

### 2.5. PR-6 Proteins

PR-6 is a class of protease inhibitors (PIs) with a molecular weight ranging from 8 to 22 kDa. These proteins contain a conserved cysteine residue in the N-terminal, which is believed to form a disulfide bond with another cysteine at the C-terminus, thereby maintaining protein structural stability [48]. Based on the specificity for pathogen-derived proteases, PR-6 proteins are classified into three major categories, including serine protease inhibitors, cysteine protease inhibitors, and aspartate proteases or metal carboxypeptidase inhibitors [49]. PR-6 belongs to the group of highly stable, naturally occurring plant defense proteins that show potent antimicrobial activity against a wide range of bacterial and fungal pathogens [50]. PR-6 binds to the target pathogen proteases to form stable complexes [51,52] that protect plant proteases from destruction. In addition to their roles in biotic stress responses, *PR-6* genes are also induced by abiotic stresses, such as low temperature [53], and by external application of phytohormones, such as jasmonic acid (JA) [54].

### 2.6. PR-7 Proteins

PR-7 proteins have a molecular weight of approximately 69 kDa and were first discovered in tomato. PR-7 has endonuclease activity and exists in a monomeric form, with its activity dependent on Ca^2+^ activation [55]. The PR-7 proteins are secreted and enriched in the extracellular space of leaf and stem cells in citrus exocortis viroid-infected tomato plants [56]. Structurally, PR-7 contains the catalytic triad, which is homologous to the *Bacillus subtilis* protease family. The catalytic triad consists of three conserved amino acid residues: Asp, His, and Ser, and it is responsible for hydrolyzing peptide bonds. PR-7 is a central β-barrel structure composed of 7–8 β-sheets, surrounded by multiple α-helices to form a stable three-dimensional conformation. The catalytic triad is located in the cleft between the β-barrel and the α-helices [57]. Functionally, PR-7 proteins have been shown to enhance the antifungal activity of plants by solubilizing the cell wall of pathogens, thereby increasing disease resistance. The induction of PR-7 proteins has been observed in response to various biotic stresses, including fungi [58] and bacteria [59], as well as abiotic stresses such as calcium deficiency [60]. Hormones like ethylene, SA, and JA [61,62] have also been implicated in the regulation of PR-7 expression.

### 2.7. PR-9 Proteins

PR-9 proteins are a class of peroxidases with a molecular weight ranging from 32–45 kDa, consisting of 10–12 conserved α-helices, 2 β-sheets, and 4 pairs of disulfide bonds [63]. PR-9 binds to the plant cell wall in two forms: an ion-bound form or a covalently bound form. PR-9 functions as a catalyst for the oxidation of substrates such as phenols and their peroxide derivatives. PR-9 plays a variety of roles in the defense of the host plant against necrotrophic or biotrophic pathogens by reinforcing cell walls and producing reactive oxygen species (ROS). In addition, PR-9 proteins are involved in seed germination, cell growth, and cell wall relaxation. Beyond their defensive and developmental functions, PR-9 proteins have demonstrated antiviral activity [64], trap heavy metals [65], and are also highly induced by a variety of environmental stresses, such as high salt and leaf tissue damage [66].

### 2.8. PR-10 Proteins

PR-10 proteins are ribonuclease-like proteins with molecular weights ranging from 16 to 19 kDa. PR-10 proteins have a similar three-dimensional structure [67] consisting of a 25-amino-acid C-terminal α-helix, a seven-stranded antiparallel β-sheets, and two N-terminal short α-helices, with a short-looped connecting sequence. The main feature of PR-10 proteins is the presence of a large Y-shaped hydrophobic lumen, a structure necessary for its transportation into the plant cell wall [68]. Most PR-10 proteins are comprised of two domains, the Bet_v_1 and P-loop domains. The Bet_v_1 domain participates in defense against pathogen infections. The P-loop domain contains a phosphate-binding loop motif that functions as a nucleotide binding site [69]. PR-10 proteins possess both antimicrobial and antiviral activities [70,71], and have been shown to play a regulatory role in plant growth, as well as in the modulation of endogenous auxin levels [72]. PR-10 proteins have been found to bind to a variety of physiological ligands, including auxins, and are implicated in plant defense responses and developmental regulation [67].

### 2.9. PR-12 Proteins

PR-12 proteins, also known as defensins, are a class of small-molecule antimicrobial peptides involved in plant defense. PR-12 proteins have at least eight highly conserved cysteine residues, which form at least four intramolecular disulfide bonds. The three-dimensional structure of PR-12 proteins includes an α-helix and an antiparallel triple-stranded β-sheet, and the main structural feature is the cysteine-stabilized α-helix β-fold motif (CSαβ) [73]. Functionally, PR-12 has been shown to possess both antifungal and antiviral properties [74]. PR-12 proteins inhibit cell–virus fusion and target viral envelopes, ultimately resulting in the lysis of viral pathogens [64,75]. Beyond their role in biotic stress responses, PR-12 has been observed to be induced by a variety of abiotic stresses, including cold [76], drought [77], and heavy metals [78].

### 2.10. PR-13 Proteins

PR-13 proteins, also known as thionins, are small cysteine-rich proteins with a molecular weight of 5 kDa. According to the three-dimensional structure, thionins can also be divided into two categories, namely α/β-thionins and γ-thionins. α/β-Thionins are composed of two α-helices and a double-stranded β-sheet. γ-Thionins are composed of one α-helix and three antiparallel β-sheets, which collectively form a two-layered α/β sandwich structure [79]. PR-13 has been shown to possess antiviral and antifungal activity. Tomato and potato plants overexpressing *PR-13* showed enhanced resistance to fungal pathogens [80]. In addition to their role in biotic stress responses, PR-13 is induced by abiotic stresses such as drought [81] and heavy metals [82].

### 2.11. PR-14 Proteins

PR-14, a class of polypeptides with a length of 90–95 amino acids, possesses the characteristics of nonspecific lipid transfer proteins (LTPs) and is thus classified as a category of LTPs. PR-14 proteins are characterized by their involvement in the process of intercellular lipid shuttle transport. The center of the three-dimensional structure of PR-14 is a bundle-like structure composed of four α- helices and a hydrophobic cavity that can accommodate multiple fluids, which facilitates the transport and loading of lipid molecules [83]. PR-14 proteins exhibit antimicrobial activity [84] and are located in the extracellular space. In addition to their defensive functions, PR-14 has been demonstrated to play a role in various plant developmental processes, including seed germination, and responses to heat and drought [85]. Furthermore, a variety of signaling molecules, including phytohormones such as ABA and SA, have been identified as regulators of PR-14 expression.

### 2.12. PR-15 and PR-16 Proteins

PR-15 and PR-16 are classified as germins (oxalate oxidase) and germin-like proteins (oxalate oxidase like), respectively, and they are members of the Calycin superfamily of proteins. PR-16 proteins contain a conserved β-barrel domain, and metal ions like Mn^2+^ and Ca^2+^ are necessary for their activity. Some PR-16 members possess superoxide dismutase (SOD) or oxalate oxidase (OXO) activity, thereby playing a role in reactive oxygen species (ROS) metabolism [86]. PR-16 proteins are resistant to proteases, distributed in the extracellular space, and generally exist in the form of heteropentamers [87]. Both PR-15 and PR-16 are glycoproteins in nature, and are produced immediately after pathogen infection [88]. PR-15 proteins play a pivotal role in defense responses against abiotic and biotic stresses by catalyzing the aerobic oxidation of oxalic acid and oxygen to produce CO_2_ and H_2_O_2_ [89]. Functionally, PR-15 and PR-16 play a part in responses to biotic stresses, such as fungi, viruses, pests, and others, as well as being involved in seed germination and floral induction [90]. PR-15 and PR-16 are responsive to abiotic stresses such as drought, low temperature, and high temperature [91,92], and the PR-16 family also plays a key role in the response of plants to saline and oxidative stresses.

### 2.13. PR-17 Proteins

PR-17 proteins are a novel class of PR proteins first identified in barley in 2002 and they play important roles in plant defense against a variety of pathogens. PR-17 proteins contain active sites and peptide-binding grooves that are highly similar to those of aminopeptidase N and Thermolysin [93]. In the amino acid sequences of PR-17 family members, there are five highly conserved domains in the central and C-terminal parts, namely Box A to Box E. The PR-17 proteins exhibit zinc affinity and structural similarity to zinc metalloproteinases [93]. The *PR-17* genes were found to be increased following treatments involving tobacco mosaic virus infection, mechanical damage, and drought conditions [94]. The overexpression of the *PR-17* gene resulted in increased tolerance to molds in wheat [95]. Finally, Table 1 summarizes the properties, molecular functions, and responses to biotic stress and abiotic stress of different PR proteins.

## 3. Response of Plant PR Proteins to Abiotic Stress

**Figure 1:**
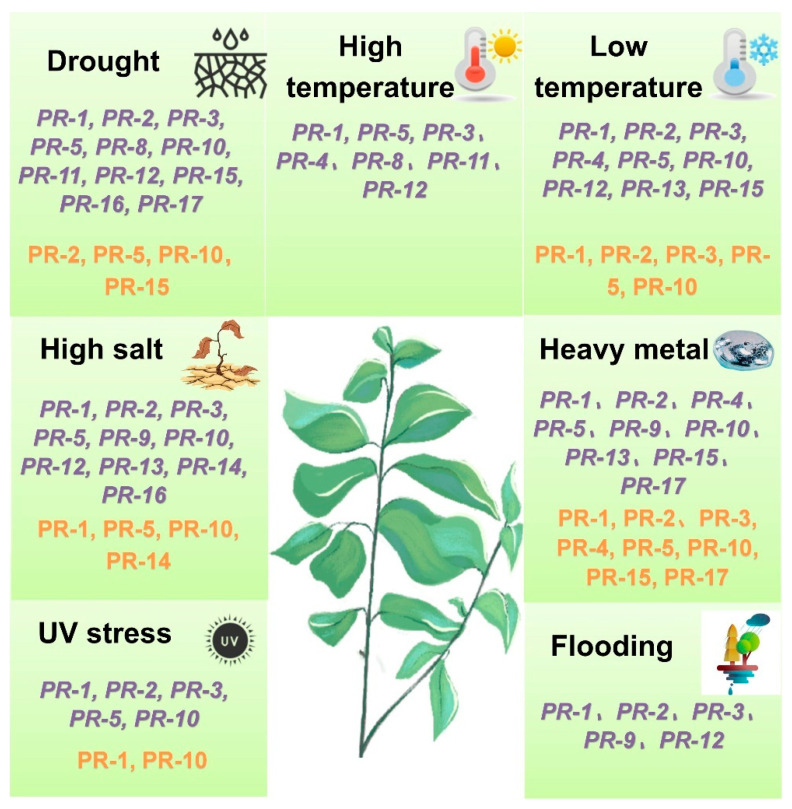
Types of PR proteins induced by different abiotic stresses. Purple italic font represents the PR protein genes; orange font represents the PR proteins.

Abiotic stress refers to adverse environmental physical or chemical factors that negatively impact plant growth, development, and survival [176]. Abiotic stress triggers significant alterations in gene expression and protein abundance within plants. Currently reported abiotic stresses that modulate the expression of plant PR genes and proteins include drought, high salt, low/high temperature, heavy metal, UV radiation, and waterlogging (Figure 1). The response of plant PR proteins to abiotic stress primarily occurs at the transcriptional level, the protein abundance level, the protein phosphorylation level, and the enzymatic activity level. Nevertheless, while transcriptional- and abundance-level changes are well-documented, there are comparatively fewer reports detailing the regulation of PR proteins at the phosphorylation and enzymatic activity levels. 

### 3.1. Drought Stress or Osmotic Stress

Under drought or osmotic stress conditions, the induction of PR families, including PR-1, PR-2, and PR-3, has been widely reported. In wheat, osmotic stress induced the expression of TaPR-1 [177]. Similarly, *PR-2* expression increased in sesame under drought stress [18]. Proteomic analysis has revealed that, in wheat, sunflower, and grapevine, PR-2, PR-5, and PR-10 proteins were significantly upregulated in response to drought stress [178]. Drought induced the expression of members from multiple PR families, including PR-3, PR-11, PR-12, PR-15, and PR-16 in *citrus* roots [59]. Drought significantly induced the expression of *PR-5* in tobacco [132]. Compared with growth under normal conditions, the transcript abundance of *PR-8* increased during drought stress in *Lotus* spp. [33]. Both the transcriptional level and protein level of *PR-10* genes were upregulated in rice under drought stress [179], accompanied by significant changes in phosphorylation status [180]. In addition, drought induced *PR-12* gene expression in pepper [77]. *PR-13* expression increased in barley plants under drought conditions [81]. PR-15 proteins levels increased in wheat flag leaves under drought stress [169]. *PR-16* transcript levels increased in soybean under drought stress [92]. The tobacco *NtPRp27*, belonging to the PR-17 family, exhibited elevated transcript levels after drought treatment [175]. Among various PR families responsive to drought stress, the PR-1 family is the most extensively studied across different plant species.

### 3.2. High Salinity Stress

Under conditions of high salinity stress, the induction of PR families including PR-1, PR-2, and PR-3 has been widely reported. For instance, *PR-1* gene expression increased in rice under high salinity stress [9], while its protein level decreased in wheat after salt stress [178]. Similarly, *PR-2* expression increased in sugarcane under high salinity [21]. *PR-3* was significantly induced by high salt stress in rice [120]. *PR-5* gene expression was upregulated in *Arabidopsis thaliana* under salt stress [181], and its protein abundance increased in soybean and wheat under salt stress [178]. *PR-9* expression was induced by salt stress in sweet potato [66]. In rice, *PR-10* expression was upregulated under salt-stress conditions [179]. PR-10 and PR-14 were accumulated in barley roots under salt stress [178]. PR-12 and PR-13 proteins levels increased in mustard seed coats subjected to salt stress treatment [162]. *PR-16* transcript levels also rose in soybean leaves after high-salinity stress [92]. Among the various PR families responsive to salt stress, the PR-10 family is the most extensively studied in plants.

### 3.3. Low Temperature Stress

Under cold stress or cold acclimation conditions, the induction of PR families, including PR-1, PR-2, and PR-3, has been reported. *PR-1* gene expression increased in tomato under low-temperature stress [102]. Similarly, *PR-2* gene expression levels increased in grapevine subjected to low-temperature treatment [113]. The chitinase, PR-2, and PR-5 protein levels rose in overwintering grasses after low-temperature treatment [113]. The abundance of several PR proteins, including PR-3 and PR-5, increased in *Ammopiptanthus mongolicus* under low-temperature treatment [182]. *PR-4* transcript levels increased in ginseng in response to low-temperature treatment [125]. *PR-10*, *PR-12*, and *PR-13* expression levels increased in wheat under low-temperature treatment [165,183]. Proteomic analysis revealed that both PR-5 and PR-10 are accumulated in winter wheat and barley after low-temperature treatment [178]. *PR-12* is similarly induced in winter wheat under low-temperature treatment [76]. *PR-15* gene expression increases in barley subjected to low-temperature stress [91]. Among the various PR families responsive to low-temperature stress, the PR-1 family is the most extensively studied in plants, followed by the PR-10 family.

### 3.4. High-Temperature Stress

Under high-temperature stress, the induction of several PR families, such as PR-1 and chitinases, has been reported. *PR-1* gene expression levels significantly increased in wheat plants after high-temperature treatment [103]. Similarly, chitinase expression increased in wheat under high-temperature conditions [36]. *PR-5* expression also increased in wheat [184] and grapevine [185] in response to heat stress. In addition, *PR-12* was induced in *A. thaliana* under high-temperature treatment [186].

### 3.5. Other Abiotic Stresses

#### 3.5.1. Heavy Metal Stress

Nine PR families, including PR-1, PR-2, and PR-4, have been reported to be induced by heavy metal stress. Proteomic analysis revealed that PR-1, PR-2, chitinase, PR-5, and PR-17 levels in barley leaf increased in response to Cd exposure [187]. Copper stress induced the expression of *PR-1* and *PR-2* genes [121,188]. Similarly, *PR-4* expression increased in maize after AgNO_3_ treatment [24]. In durum wheat, *PR-5* was strongly induced by several heavy metals, such as Cd, Cu, and Zn [136]. Four defensin genes were induced by Zn exposure, but not by Cd exposure in *A*. *thaliana* [78]. The expression of *PR-9* increased under heavy metal stress in tomato [189], and its enzymatic activity increased in water lily under similar conditions [65]. PR-10 proteins were accumulated in yellow lupin root tips after lead ion treatment, and *PR-10* gene transcriptional activity increased under Pb, Cd, and As treatments [153]. Similarly, PR-10 proteins were upregulated in Cd-treated flax cells [178]. Copper stress induced increased PR-10 proteins expression in both the roots and leaves of copper/zinc-tolerant birch [190]. *PR-13* gene expression increased in rice under Cd exposure [82]. In addition, Cd, Co, and Cu significantly induced PR-15 content in wheat plants [191]. Among the various PR families responsive to heavy metal stress, the PR-10 family is the most extensively studied in plants.

#### 3.5.2. UV Radiation Stress

Under UV-B stress, the induction of families such as PR-1, PR-2, and PR-3 has been observed, involving responses at the gene transcription and protein accumulation levels. In tobacco, UV-B induced PR-1 with specific accumulation in irradiated leaf areas [105]. UV-B increased the transcript levels of *PR-1*, *PR-2*, and *PR-5* in *A*. *thaliana* [115]. Similarly, PR-2 was significantly induced in grapevine in response to UV radiation [113]. The transcript levels of *PR-3* and *PR-5* in UV-treated strawberry leaves were significantly higher than those in non-irradiated controls [192]. PR-10 proteins increased in the leaves of white lupin after UV treatment [154]. Among the various PR families responsive to heavy metal stress, the PR-10 family is the most frequently analyzed in plants.

#### 3.5.3. Waterlogging Stress

Under waterlogging stress, the induction of several families such as PR-1, PR-2, and PR-3 has been reported at the gene transcription or protein accumulation levels. The expression level of *PR-1* significantly increased in Vietnamese snowbell under waterlogging stress [193]. Similarly, *PR-2* and *PR-9* expressions were upregulated in soybean and maize under waterlogging stress [178]. Transcriptomics analysis revealed that transcript levels of PR-3 increased in the leaves and roots of waterlogged wheat, specifically involving class IV and class VIII chitinases [194]. In addition, *PR-12* gene expression increased in onion leaves when plants were subjected to waterlogging stress [195].

Overall, among the various PR families, PR-1 is the most frequently reported PR family involved in abiotic stress responses. Studies on changes in the gene expression levels of various PR families under stress are more numerous than studies at the protein level. Among the different abiotic stressors influencing PR levels, drought stress has been the most extensively studied.

## 4. Regulation of PR Proteins Involved in Abiotic Stress Responses

**Figure 2:**
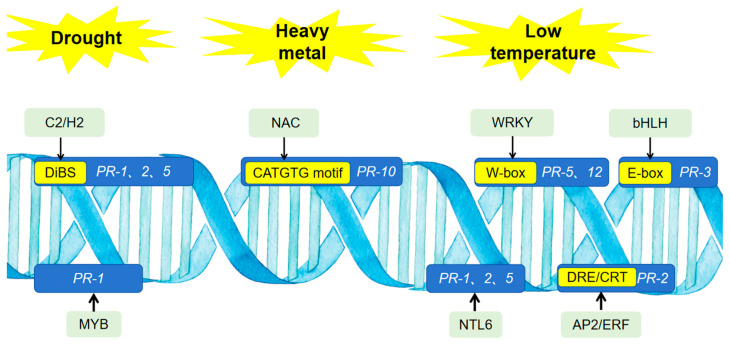
Transcription factors directly regulate *PR* genes under abiotic stress. The text within green boxes denotes transcription factor families that regulate the expression of *PR* genes under abiotic stress conditions. The text in blue boxes represents *PR* genes. Yellow boxes contain sequences of cis-regulatory elements in the promoter regions of *PR* genes that are bound by transcription factors. Arrows indicate that the transcription factor family can bind to the corresponding cis-regulatory element of the *PR* genes.

PR proteins, as key components of the plant defense system, play crucial roles in mediating responses to both biotic and abiotic stresses. While the above sections describe significant alterations in *PR* gene expression and protein abundance under abiotic stress conditions, the signaling transduction pathways by which abiotic stress signals regulate *PR* genes or proteins remain poorly understood. Notably, abiotic stress signaling pathways may utilize components of biotic stress signaling pathways; therefore, this section first briefly introduces the signaling pathways regulating *PR* genes under biotic stress (Figure 2).

Extensive research has revealed that pathogen signals activate *PR* gene expression primarily through hormone signaling pathways such as SA and JA. However, PR proteins are not regulated by a single hormone alone, but function through complex crosstalk involving multiple stress hormones and their signaling components—such as SA (via NPR1), JA (via MYC2), and ethylene (via ethylene response factor)—alongside synergistic or antagonistic interactions with growth hormones like gibberellin and cytokinin. This dynamic network balances defense and growth, reflecting the multidimensional complexity of the plant stress responses rather than a simple summation of individual pathways [159]. In this process, transcription factors, including the bZIP family factor TGA, the NAC family factor ATAF2, and the AP2/ERF family factor EREBP1, play important roles in the transcriptional activation of PR genes. The SA signaling pathway relies on the coactivator NPR1 interacting with the bZIP transcription factor TGA. This interaction promotes TGA binding to the LS7 cis-element in the *PR-1* promoter, thereby activating *PR-1* transcription and mediating system-acquired resistance (SAR) [196]. The expression of the *Arabidopsis* NAC transcription factor ATAF2 is induced by mechanical wounding, SA, and MeJA. ATAF2 binds to the promoters of *PR-1*, *PR-4*, *PR-12*, and other *PR* genes, repressing their transcription [197]. In rice, the MAPK cascade kinase BWMK1 integrates signals from fungal elicitors, H_2_O_2_, SA, and JA. BWMK1 translocates to the nucleus via its C-terminal domain, phosphorylates the AP2/ERF transcription factor OsEREBP1, thereby enhancing its binding affinity to the GCC-box cis-element (AGCCGCC) in *PR* genes promoters, and coordinately activates the expression of *PR-1*, *PR-2*, *PR-5*, and other *PR* genes, conferring broad-spectrum resistance to fungi and bacterial pathogens [198].

Under abiotic stress conditions, the transcriptional regulatory mechanisms governing plant PR proteins are not fully elucidated. Promoter analysis indicates that the promoter regions of various plant *PR* genes contain multiple cis-acting elements associated with both biotic and abiotic stress responses, harboring binding sites for transcription factors such as WRKY, HD-Zip, AP2/ERF, and MYB. In soybean, predicted transcription factor family binding sites in the *PR-1* gene promoter include members of the WRKY, GATA, HD-Zip, ERF, and C2H2 families [101]. Similarly, the rice *PR-1* gene promoter is predicted to harbor binding sites for WRKY, bZIP, AP2/ERF, bHLH, MYB, TCP, SBP, and B3 transcription factors [199].

Experimental evidence supports the involvement of several transcription factor families, including C2H2, NAC, WRKY, and MYB, in mediating the regulation of *PR* genes expression by abiotic stress signals such as drought, low temperatures, and heavy metal exposure. Under drought conditions, the *Arabidopsis* transcription factor Di19 (Drought-induced), a C2H2 zinc finger protein, activates the expression of *PR-1*, *PR-2*, and *PR-5* genes by binding to the conserved sequence TACA (A/G)T within their promoters [10]. The *Arabidopsis* Di19 gene family encodes seven hydrophilic proteins, with Di19 being one of them, containing two atypical C2H2-type zinc finger domains and a nuclear localization signal (NLS). A drought-induced cotton R2R3-MYB transcription factor, GhMYB36, binds to the *PR-1* promoter and upregulates PR-1 expression [200].

Under cold stress conditions, the *Arabidopsis* NAC transcription factor NTL6 directly binds to and activates the expression of *PR-1*, *PR-2*, and *PR-5* genes [11]. NTL6 belongs to membrane-bound transcription factors containing an N-terminal NAC domain (DNA-binding domain) and a C-terminal transmembrane motif. A cold-stress-responsive *Arabidopsis* ERF/AP2 transcription factor, DREB1, induces *PR-2* gene transcription by binding to the DRE/CRT element (GCCGAC) within its promoter [201]. Under low-temperature and osmotic stress, *A*. *mongolicus* transcription factor AmWRKY14 directly binds to the W-box cis-element of the *PR-12* gene promoter and activates its expression [202]. Under cold, drought, and wounding stress, the jojoba transcription factor ScWRKY41 promotes *PR-5* expression by binding to W-box elements in its promoter region [203]. Similarly, under low-temperature stress, ScMYC2, a bHLH family member in jojoba, regulates *PR-3* expression by binding to the E-box cis-element in its promoter [204].

Under heavy metal stress, the rice transcription factor OsNAC300, a NAC transcription factor, directly binds to the CATGTG motif in the promoter region of *PR-10* genes and activates their transcription [205].

Compared with well-characterized regulatory pathways involved in *PR* gene activation under biotic stress, the regulatory mechanisms underlying PR gene regulation under abiotic stress remain less defined. However, some experimental evidence exists. For instance, within the bHLH transcription factor family, it was found that low temperature increases the JA content in leaves. JA acts as a signaling molecule, initiating a signal transduction pathway where the transcription factor MYC2 binds to specific cis-elements in the promoter regions of target genes to regulate their expression [204].

## 5. Biological Functions of PR Proteins in Abiotic Stress Responses

Previous sections summarized the induction of *PR* genes expression under abiotic stress conditions and the transcriptional regulatory mechanism involved. How do PR proteins function under abiotic stress conditions? Given that different PR families possess distinct conserved domains, PR proteins within a single PR family typically exhibit similar molecular activities. Below, we detail the specific biological functions of PR-1 to PR-17 in the context of abiotic stress responses.

The overexpression of *PR-1* enhanced tolerance to drought and salt stress in transgenic *Arabidopsis* [9] and cold tolerance in transgenic *chrysanthemum* [206]. Several mechanisms have been proposed through which PR-1 may help plants to tolerate abiotic stress: (1) PR-1 helps to maintain plasma membrane stability. The CAP superfamily domain in PR-1 proteins may regulate membrane stability under stress conditions by binding sterols. (2) PR-1 forms chimeric receptor proteins: The fusion of PR-1 with receptor-like protein kinases (RLKs) potentially generates novel proteins that perceive extracellular signals, undergo conformational changes, and activate intracellular phosphorylation cascades in response to extracellular cues [207]. Additionally, PR-1 may indirectly confer tolerance by modulating antioxidant enzyme activity [206] and regulating stomatal aperture [10].

Transgenic *Arabidopsis* plants overexpressing *PR-2* (β-1,3-glucanase) showed increased aluminum tolerance [208], while other studies suggest that PR-2 accumulation may contribute to enhancing low-temperature tolerance in *citrus* [209]. PR-2 may play a part in abiotic stress responses in the following ways: (1) PR-2 helps to protect thylakoid membranes. Tobacco PR-2 protects spinach thylakoids from freeze-thaw induced damage [20]. (2) PR-2 proteins function as ice-binding proteins (IBPs). Winter rye PR-2 specifically binds ice crystals, inhibiting their growth and recrystallization, thereby conferring freezing tolerance [19]. (3) PR-2 proteins hydrolyze carbohydrates. Winter rye PR-2 hydrolyzes β-glucan polymers, generating small soluble carbohydrates that increase cellular solute concentration and depress freezing points. (4) PR-2 proteins form synergistic complexes. PR-2 complexes with other antifreeze proteins (e.g., chitinases, PR-5) exhibit significantly higher activity in modifying carbohydrates and controlling ice crystal morphology than individual PR-2 [19].

PR-3, PR-4, PR-8, and PR-11 belong to the chitinase family. Chitinase overexpression enhanced tolerance to low temperature and osmotic stress in transgenic *Arabidopsis* and jojoba [204], as well as to salt and heavy metal stress in tobacco [188]. These proteins may contribute to abiotic stress tolerance through two primary mechanisms: (1) Chitinases indirectly promote plant tolerance via enhancing antioxidant enzyme activity. They reduce malondialdehyde (MDA) accumulation and effectively scavenge reactive oxygen species (ROS), thereby mitigating oxidative damage induced by stress like drought. (2) Chitinases participate in stress responses by promoting chitosan oligosaccharide (COS) production. Chitinases in the GH18 subfamily, dependent on the DxDxE motif, are proposed to generate COS, which participates in antioxidation and osmoregulation. Notably, higher COS polymerization degrees are correlated with stronger antioxidant activity in barley [210].

The overexpression of *PR-5* (thaumatin-like proteins, TLPs) enhanced drought and salt tolerance in transgenic tobacco, rice, and *Arabidopsis* [132,181,211]. The first direct way in which PR-5 helps plants to tolerate stress is Na^+^ sequestration. Under high salinity, tobacco PR-5 binds to Na^+^ channels on the plasma membrane and coordinates Na^+^ ions, sequestering and compartmentalizing them into the apoplast and vacuoles to regulate osmotic potential [212]. The second direct way in which PR-5 helps plants to tolerate stress is by modulating cytoskeleton dynamics. Potato PR-5 stabilizes actin filaments (AFs) by direct binding and blocks Ca^2+^ channel activity, preventing cytosolic Ca^2+^ elevation and enhancing cold tolerance [213]. The third direct way in which PR-5 helps plants to tolerate stress is by protecting the plant cell membrane. Tobacco PR-5 interacts with membrane lipids and competitively binds lipid peroxidation products, thereby reducing oxidative damage under drought and salt stress [214]. Rice PR-5 acts as a molecular chaperone, interacting with membrane proteins to prevent their denaturation and membrane disintegration under drought conditions [215]. The fourth way in which PR-5 helps plants to tolerate stress is the fusion with kinases to form transmembrane receptors. The *Arabidopsis* chimeric receptor PR5-RLK contains an extracellular PR-5-like domain, transmembrane domain, and intracellular serine/threonine kinase domain, which are induced by drought conditions. Under stress conditions, PR5-RLK phosphorylates downstream transcription factors and ion channel proteins, inducing drought-responsive gene expression and stomatal closure [216,217]. In addition, PR-5 can indirectly participate in plant stress tolerance in three different ways, including increasing the thickness of secondary cell walls [120], promoting proline synthesis via the ABA signaling pathway [218,219], and enhancing enzyme activity such as antioxidant enzymes [211,215].

Studies suggest that several PR-9 proteins (peroxidases) may help to enhance plant heavy metal tolerance. Water lily utilizes PR-9 to produce phenolic polymers that capture cadmium (Cd), sequestering it as Ca–Cd crystals in specific glands on the leaf water surface [65]. Similarly, the overexpression of PR-13 (thionin) enhanced Cd tolerance in transgenic rice [82]. Under Cd stress, rice PR-13 localizes to the cell wall, significantly increasing Cd binding to the wall, thereby reducing Cd upward translocation and accumulation in shoots and straw [82].

Overexpression of *PR-10* (ribonuclease-like proteins) genes has been shown to enhance salt and drought tolerance in rice and the halophyte *Halostachys capsica* [152,179]. PR-10 indirectly improves stress tolerance by increasing the activity of the plant antioxidant enzyme system and regulating osmoprotectant synthesis to effectively scavenge ROS, thereby protecting cells from oxidative damage [179].

Transgenic *Arabidopsis* overexpressing *PR-14* (LTP) exhibited enhanced tolerance to salt and drought stress [220]. PR-14 interacts with cellular membranes, regulating membrane lipid fluidity and permeability. Under drought, salt, or low temperature, the hydrophobic cavity of PR-14 binds membrane lipids, stabilizing membrane structure and preventing lipid peroxidation. In addition, PR-14 also modulates membrane composition, participates in cuticle and suberin biosynthesis, and facilitates the transport of hydrophobic lipid precursors to the cell wall, thereby strengthening the epidermal barrier function [166].

The accumulation of PR-15 (oxalate oxidase) may be beneficial for plant tolerance to waterlogging, high-temperature, and high-salinity conditions. PR-15 functions through both OXO and SOD activities, and it catalyzes oxalate degradation to produce H_2_O_2_, which may participate in cell wall lignification, enhancing wall stability and helping plants to withstand high-temperature damage to cellular structures [221]. Moreover, H_2_O_2_ acts as a signaling molecule, activating defense mechanisms enabling plant adaptation to waterlogging stress [170]. Furthermore, the Mn^2+^/Mn^3+^-dependent SOD activity of moss PR-15 scavenges excess intracellular superoxide radicals, mitigating oxidative damage and protecting cells from high salinity [222].

Transgenic *Arabidopsis* overexpressing *PR-16* (oxalate oxidase-like proteins) showed enhanced tolerance to drought and salt stress [92]. PR-16 in moss and soybean exhibits SOD activity [222], thereby protecting chloroplasts by scavenging ROS and maintaining ion homeostasis. Additionally, PR-16 enhances stress tolerance by regulating the accumulation of osmoprotectants and inducing stomatal closure to reduce water loss under drought and salt stress [92].

**Figure 3:**
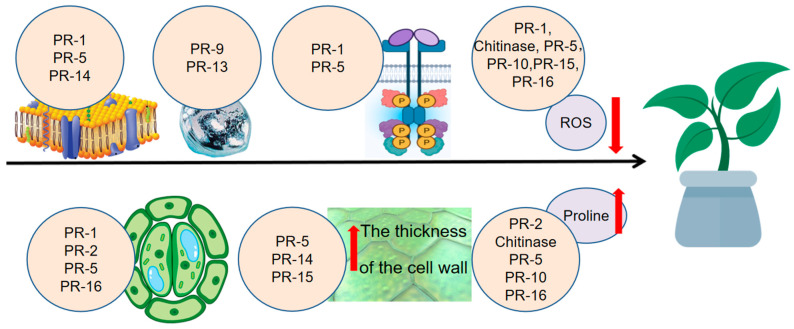
Mechanism of PR proteins enhancing plant abiotic stress tolerance. PR-1, PR-5, and PR-14 enhance plant tolerance to abiotic stress by directly binding to membrane lipids or proteins, thereby increasing membrane stability. PR-9 and PR-13 mitigate heavy metal stress through direct binding to metal ions. PR-2 and PR-5 contribute to abiotic stress tolerance through the formation of fusion proteins with receptor kinases. PR-1, chitinases (PR-3, 4, 8, 11), PR-5, PR-10, PR-15, and PR-16 confer indirect tolerance by enhancing the plant’s antioxidant capacity; the specific mechanism of PR-1, chitinases (PR-3, 4, 8, and 11), PR-5, and PR-10′s actions remain currently unclear. PR-1, PR-2, PR-5, and PR-16 reduce stomatal aperture to indirectly alleviate abiotic stress. PR-2, chitinases (PR-3, 4, 8, and 11), PR-5, PR-10, and PR-16 modulate osmotic potential through the accumulation of soluble compounds. PR-5, PR-14, and PR-15 fortify cell walls to indirectly enhance stress tolerance. Abbreviations: PR, pathogenesis-related protein; ROS, reactive oxygen species.

In summary, PR families contribute to abiotic stress tolerance through diverse mechanisms (Figure 3). The first one is directly enhancing membrane stability via binding lipids/membrane proteins (PR-1, PR-5, and PR-14). The second is directly binding metal ions to participate in heavy metal tolerance (PR-9 and PR-13). The third one is indirectly enhancing antioxidant capacity, and this mechanism involves PR-1, Chitinases (PR-3, 4, 8, 11), PR-5, PR-10, PR-15, and PR-16. The fourth one is indirectly reducing stomatal aperture (PR-1, PR-2, PR-5, and PR-16). The fifth way is indirectly regulating osmotic potential via soluble compounds accumulation, and this mechanism involves PR-2, Chitinases (PR-3, 4, 8, 11), PR-5, PR-10, and PR-16. The sixth way is indirectly increasing cell wall thickness, and this mechanism involves PR-5, PR-14, and PR-15.

Some PR families rely on their enzymatic activity to help plants to tolerate abiotic stress, including PR-2 (β-1,3-glucanase), PR-3, 4, 8, and 11 (chitinases), PR-9 (peroxidase), PR-10 (ribonuclease), PR-15 (oxalate oxidase), and PR-16 (oxalate oxidase-like protein) (Table 2). These PR families directly or indirectly participate in stress responses through catalytic reactions. While other PR families use their specific three-dimensional structures to help plants to tolerate abiotic stress, including PR-1, PR-5, PR-13, and PR-14, these PR families rely on the three-dimensional structure of proteins, such as binding sites and membrane interaction regions, to exert membrane stability, ion sequestration, and other functions.

## 6. Conclusions

### 6.1. Critical Knowledge Gaps

Research on PR proteins in the context of abiotic stress responses holds significant scientific importance and application value. A major critical knowledge gap lies in the lack of a clear distinction between the direct and indirect roles of PR proteins in abiotic stress responses, which has hindered their systematic study in this context. Evolutionarily conserved as core components of biotic stress defense, PR proteins are often induced under abiotic stress through secondary mechanisms—such as crosstalk with general stress signaling networks (e.g., SA-ABA interplay, ROS-mediated pathways) or overlap with shared transcriptional regulators (e.g., WRKY, ERF/AP2 factors)—rather than via dedicated abiotic stress-specific pathways. This indirect involvement, coupled with historical research focus on their biotic defense functions, has limited in-depth exploration of their abiotic roles. Further, the mechanistic basis for why PR proteins are induced under abiotic stresses remains poorly synthesized. While transgenic overexpression of *PR* genes can enhance abiotic tolerance, this is often attributed to pleiotropic effects, rather than direct functional roles. Additionally, the ecological relevance of such induction—for instance, whether it primarily serves to preempt pathogen colonization under abiotic stress-weakened states, or reflects genuine adaptation to abiotic stress—lacks systematic analysis. Finally, current studies largely report *PR* expression patterns under abiotic stresses without integrating these observations into a unifying framework. This gap limits our understanding of whether PR proteins act as “stress integrators” (linking biotic and abiotic responses) or merely as byproducts of overlapping stress pathways. Although the mechanisms by which specific PR protein families confer abiotic stress tolerance to plants are partially understood, critical knowledge gaps persist regarding the molecular mechanisms underlying the involvement of most PR families. For instance, it has been clarified that PR-16, which has SOD activity, can scavenge ROS in plants and enhance their antioxidant capacity. In contrast, the mechanisms by which PR-1, PR-3, PR-4, and other families enhance plant antioxidant enzyme activity remain largely unknown. Similarly, the molecular basis of stomatal aperture regulation by PR-1, PR-2, and PR-5 has yet to be elucidated.

Although upstream transcription factors and signaling pathways regulating specific PR genes are known for some individual PR proteins, the precise molecular pathways governing the regulation of most *PR* genes by abiotic stress signals remain largely obscure. In particular, the coupling mechanisms between upstream transcription factors (e.g., WRKY and NAC) and abiotic stress signals are not fully revealed, particularly concerning the synergistic regulatory networks involving hormone pathways (SA, JA, and ABA) and PR genes. Moreover, current research on the impact of post-translational modifications (PTMs) (e.g., phosphorylation and glycosylation) on PR proteins activity and stability is rather limited. For example, the structural basis by which phosphorylation enhances the ribonuclease activity of PR-10 remains unelucidated.

Systematic studies on the functional specificity and cross-regulatory mechanisms of PR proteins under combined abiotic stresses (e.g., drought + heat, salinity + heavy metals) are still lacking, and how they balance stress tolerance with growth and development requires deeper investigation. The mechanisms underlying functional redundancy and functional divergence within PR families are unclear. For instance, the distinct roles and potential synergistic interactions of different classes of chitinases belonging to PR-3, PR-4, PR-8, and PR-11 families in abiotic stress responses need further delineation.

The evolutionary trajectories and functional innovations of PR families in extremophile plants have not been systematically investigated, and the mining of stress-resistance gene resources remains largely confined to model plants. In addition, functional studies on PR-like proteins in lower plants (e.g., bryophytes and algae) are virtually non-existent, leaving the evolutionary process of their primordial defense mechanisms adapting to abiotic stresses largely unexplored.

### 6.2. Prospects for Study on PR Proteins

Bioinformatics approaches should be employed to conduct systematic evolutionary analyses of individual PR families across the plant kingdom. This will identify the full repertoire of PR families from lower to higher plants, clarify their evolutionary paths, define the structural changes occurring at specific evolutionary junctures, and elucidate the acquisition of novel functions. Special focus should be placed on understanding how PR proteins transitioned from classical defense roles against biotic stresses to functions in abiotic stress tolerance. In addition, the exploitation of *PR* gene resources in extremophile plants should be strengthened. PR homologous genes in desert plants and halophytes should be systematically screened, and the association between their structural features and abiotic stress tolerance should be analyzed.

Multi-dimensional technologies should be combined with spatial omics to reveal dynamic expression patterns of PR proteins in specific tissues upon abiotic stress treatment. Structural biology approaches and AI prediction should be leveraged, with cryo-electron microscopy and machine learning employed to resolve high-resolution three-dimensional structures of PR proteins and design functionally enhanced mutants. Post-translational modification landscapes of PR proteins should be deciphered using phosphoproteomics and ubiquitinomics analyses to elucidate regulatory effects on PR protein stability and interaction networks under stress conditions.

The increasing frequency of extreme weather events due to global climate change should be addressed through transgenic approaches utilizing PR proteins to develop crops tolerant to both biotic and abiotic stresses. Gene editing and synthetic biology should be prioritized to investigate multi-gene synergistic regulation. In particular, CRISPR-Cas9 technology should be employed for the targeted editing of multiple PR family genes to overcome functional redundancy while enhancing broad-spectrum stress resistance. The design and application of stress-specific promoters should be developed to drive high-efficiency expression of *PR* genes under defined stress conditions. Concurrently, crop quality traits should be optimized through PR gene editing to achieve synergistic improvement of stress tolerance and agronomic yield.

The yield performance and stress tolerance stability of *PR* transgenic crops should be evaluated under field conditions in realistic agricultural settings (e.g., drought and saline–alkaline soils), and phenotype–genotype association databases should be established. Furthermore, the impact of PR overexpression on soil microbiomes and ecosystem health should be assessed to ensure the environmental safety of transgenic crops. Environmental remediation applications should be explored by exploiting the pollutant-degrading capacity of PR-9 peroxidases, with phytoremediation technologies developed for soils contaminated with heavy metals and organic pollutants. Innovative nanocarrier-mediated delivery systems should be investigated through the design of PR protein nanoparticles for foliar application, enabling rapid activation of plant stress responses while reducing reliance on genetic modification. Finally, artificial intelligence should be utilized to construct predictive models of PR protein functioning, thereby accelerating intelligent molecular breeding for stress-tolerant crops.

## Figures and Tables

**Table 1:** Classification, molecular function, and stress response of PR proteins.

	Property	Molecular Function	Biotic Stress	Abiotic Stress
PR-1	Antifungal	Antimicrobial activity [96] Pathogen toxin degradation Viral coat protein-receptor binding inhibition [8] Recruitment of PR proteins (PR-5/PR-14) [97] Sterol binding [98]	Bacteria [99] Fungi [100] Viruses [8] Oomycetes Insects Nematodes [101]	Drought [12] High salinity [9] Low temperature [102] High temperature [103] Heavy metals [104] UV radiation [105]
PR-2	β-1,3-glucanase	Microbial growth suppression [106] Fungal cell wall degradation [14] Callose deposition modulation [107]	Bacteria [108] Fungi [14] Oomycetes [109] Viruses [110,111] Nematodes [112]	Drought [18] High salinity [21] Low temperature [113] Heavy metals [114] UV radiation [115]
PR-3	Endochitinase (Classes I, II, IV, V, VI, VII)	Chitinase activity [116] Fungal cell wall decomposition [117]	Fungi [118] Viruses [119] Nematodes [112]	Drought [59] High salinity [120] High temperature [36] Heavy metals [121]
PR-4	Endochitinase (Classes I, II)	Spore germination inhibition Hyphal growth suppression Cell wall degradation assistance Ribonuclease/DNase activity [28,122]	Fungi [123] Insect and pests [28]	Drought [124] High salinity [23] Low temperature [125] Heavy metals [24]
PR-5	Thaumatin-like protein	Microbial growth inhibition [106] Fungal membrane permeabilization Membrane potential dissipation [126] Fruit ripening promotion [127]	Fungi [128] Viruses [129,130] Bacteria [131] Nematodes [112]	Drought [132] High salinity [133] Osmotic stress [46] Low temperature [134] High temperature [135] Heavy metals [136] UV radiation [115]
PR-6	Protease inhibitor	Pathogen protease inhibition Host cell degradation prevention [137]	Bacteria, Fungi [137]	Low temperature [53]
PR-7	Alkaline endoprotease	Fungal structural proteins degradation Cell wall integrity impairment [138]	Fungi [58] Bacteria [59] Viruses [139] Nematodes [140]	-
PR-8	Class III chitinase	Bacterial cell wall hydrolysis [123]	Fungi [141]	Drought [33]
PR-9	Peroxidase	ROS concentration modulation Lignin-mediated cell wall reinforcement Cytotoxic radical production [142]	Fungi [143] Bacteria [144] Viruses [64]	Drought [145] High salinity [66] Heavy metals [65]
PR-10	Ribonuclease-like proteins	Ribonuclease activity [70] Small hydrophobic ligand binding [146] Fungal/bacterial growth inhibition [147] Secondary metabolite biosynthesis regulation [148] Stress response participation Iron chelation [149] Growth/development promotion [150]	Viruses Bacteria [151] Fungi Nematodes Insects [147]	High salinity Drought [152] Heavy metals [153] Low temperature [70] UV radiation [154]
PR-11	Class I chitinase	β-1,4-chitin glycosidic bond hydrolysis Hyphal structure disruption Spore germination inhibition [155] Pathogen defense enhancement [123]	Bacteria [59] Viruses [156]	Drought [59]
PR-12	Plant defensin	Antimicrobial activity Systemic defense potentiation [157] Human IgE-mediated allergenicity [158] Membrane pore formation Pathogen protease inhibition [159]	Fungi [160] Viruses [64] Nematodes [161]	Drought [77] High-salt/high-temperature stress [162] Low temperature [76] Heavy metals [78]
PR-13	Thionin	Phospholipid binding Transmembrane pore formation Ion leakage induction [163] Damage-associated molecular pattern function [1]	Bacteria [164] Fungi [80] Viruses [64]	Drought [81] High-salt/high-temperature stress [162] Low temperature [165] Heavy metals [82]
PR-14	Lipid transfer proteins	Lipid binding/transport Membrane biosynthesis participation Pathogen membrane disruption [166] Lipid signaling modulation [166,167] SAR network synergy [81]	Bacteria Fungi [84] Viruses [64]	Drought High salinity Low temperature [166]
PR-15	Oxalate oxidase	ROS-controlled antimicrobial activity [168]	Fungi [88] Bacteria [59] Viruses Insect and pests [90]	Drought [169] Low temperature [91] Heavy metals [86] High temperature High salinity Waterlogging [170]
PR-16	Oxalate oxidase-like	Fungal toxin inhibition [171] SOD-mediated superoxide scavenging Defense gene activation [92,172] H_2_O_2_-dependent cell wall fortification [173] ABA sensitivity enhancement [92] Pathogen wall hydrolysis [86]	Fungi [88] Bacteria Viruses [59]	Drought [59] High salinity [174]
PR-17	Antifungal and antiviral	Extracellular protease activity [93]	Viruses Fungi [94] Bacteria [59]	Drought [175]

**Table 2:** PR Families with enzymatic activity.

PR Family	Enzymatic Activity Type	Biological Functions
PR-2	β-1,3-Glucanase	Hydrolysis of β-1,3-glucans in fungal cell walls Release of elicitors Carbohydrate metabolism Osmotic adjustment
PR-3, 4, 8, 11	Chitinase	Degradation of chitin (fungal cell walls/insect exoskeletons) Partial lysozyme activity Antioxidant response COS generation
PR-9	Peroxidase	H_2_O_2_-dependent oxidative burst catalysis Lignification promotion ROS scavenging Phenolic compound metabolism
PR-10	Ribonuclease	RNA degradation Signal transduction Phytohormone binding Modulation of antioxidant enzyme activities
PR-15	Oxalate oxidase	Oxalate degradation with H_2_O_2_ generation Cell wall lignification ROS signaling
PR-16	Oxalate oxidase-like protein	SOD activity ROS scavenging Ion homeostasis maintenance

## Data Availability

No new data were created or analyzed in this study.
